# Combined whole cell wall analysis and streamlined in silico carbohydrate-active enzyme discovery to improve biocatalytic conversion of agricultural crop residues

**DOI:** 10.1186/s13068-020-01869-8

**Published:** 2021-01-09

**Authors:** Jeffrey P. Tingley, Kristin E. Low, Xiaohui Xing, D. Wade Abbott

**Affiliations:** 1 Lethbridge Research and Development Centre, Agriculture and Agri-Food Canada, 5403-1st Avenue South, Lethbridge, AB T1J 4B1 Canada; 2 Department of Biochemistry, University of Lethbridge, Lethbridge, AB T1K 6T5 Canada

**Keywords:** Agriculture, Crop residues, Biomass conversion, Carbohydrate-active enzyme, Plant cell wall, Glycosidic linkage analysis, Functional genomics, Phylogeny

## Abstract

The production of biofuels as an efficient source of renewable energy has received considerable attention due to increasing energy demands and regulatory incentives to reduce greenhouse gas emissions. Second-generation biofuel feedstocks, including agricultural crop residues generated on-farm during annual harvests, are abundant, inexpensive, and sustainable. Unlike first-generation feedstocks, which are enriched in easily fermentable carbohydrates, crop residue cell walls are highly resistant to saccharification, fermentation, and valorization. Crop residues contain recalcitrant polysaccharides, including cellulose, hemicelluloses, pectins, and lignin and lignin-carbohydrate complexes. In addition, their cell walls can vary in linkage structure and monosaccharide composition between plant sources. Characterization of total cell wall structure, including high-resolution analyses of saccharide composition, linkage, and complex structures using chromatography-based methods, nuclear magnetic resonance, -omics, and antibody glycome profiling, provides critical insight into the fine chemistry of feedstock cell walls. Furthermore, improving both the catalytic potential of microbial communities that populate biodigester reactors and the efficiency of pre-treatments used in bioethanol production may improve bioconversion rates and yields. Toward this end, knowledge and characterization of carbohydrate-active enzymes (CAZymes) involved in dynamic biomass deconstruction is pivotal. Here we overview the use of common “-omics”-based methods for the study of lignocellulose-metabolizing communities and microorganisms, as well as methods for annotation and discovery of CAZymes, and accurate prediction of CAZyme function. Emerging approaches for analysis of large datasets, including metagenome-assembled genomes, are also discussed. Using complementary glycomic and meta-omic methods to characterize agricultural residues and the microbial communities that digest them provides promising streams of research to maximize value and energy extraction from crop waste streams.

## Background

Growing international concern over climate change has led to continued interest in generating bioliquids (*e.g.,* ethanol) and biogases (*e.g.,* methane) from viable and sustainable sources of energy. First-generation biofuel crops, such as corn and sugarcane, which contain high amounts of starch and sucrose, respectively, are readily fermented by microorganisms to produce ethanol and biogas in biodigesters [1, 2]. However, their use for biofuel production has socioeconomic consequnces , including the food versus fuel debate, as their dedicated use for fuel directly impacts food prices and competition of land usage [3]. Second-generation biofuel crops do not compete directly with food production and have been well regarded as sustainable sources of fermentable biomass. These feedstocks include inedible woody plants, bioenergy crops (*e.g.,* switchgrass), and agricultural residues.

Crop residues are biomaterials remaining in the field after harvest and consist mainly of straw or stover from grains and oilseeds. Primary sources include rice (*Oryza sativa*), wheat (*Triticum aestivum*), corn (*Zea mays*), barely (*Hordeum vulgare*), oat (*Avena sativa*), rye (*Secale cereale*), canola (*Brassica napus*), flax (*Linum usitatissimum*), peanut (*Arachis hypogaea*), sunflower (*Helianthus annuus*), sorghum (*Sorghum bicolor*), soybean (*Glycine max*), pea (*Pisum sativum*), and chickpea (*Cicer arietinum*) [4–12]. Historically, crop residues are usually left to decay on field after threshing and were incorporated into soil by plowing and disking or used as livestock feed or bedding [13]. Seasonal burning of agricultural residues is practiced in many countries, resulting in large scale wastage and has been linked to environmental problems, such as emission of airborne particulate matter (PM) pollutants (*e.g.,* PM_2.5_) and greenhouse gases [14, 15].

Crop residues are readily available and produced in great quantities. Globally, the total residue produced from a collection of 27 common food crops was estimated to be 3.8 billion tonnes per year [16], and the theoretical global energy potential from six major crop residues was estimated to be 65 exajoules per year, equaling 66% of annual worldwide transportation energy consumption in 2006–2008 [7]. However, the high concentration of lignocellulosic biomass, including recalcitrant polysaccharides, such as cellulose, hemicelluloses, pectins, and aromatic polymers (*i.e.,* lignin), has limited their widespread use in biofuel production. Cross-linking of hemicellulose to lignin and hemicellulose–cellulose interactions further contribute to biomass recalcitrance [17]. Moreover, the diversity of monosaccharide composition and non-cellulosic carbohydrate lignin linkages can vary between crop residues [18], affecting their valorization as high-value products, including ethanol and methane.

Carbohydrate-active enzymes (CAZymes) are commonly used in biofuels to convert recalcitrant polysaccharides into fermentable carbohydrates. In bioethanol production, CAZymes are added to biomass prior to or simultaneously with fermentation, or expressed from an engineered organism for consolidated bioprocessing [19]; whereas biogas production uses the native production of CAZymes from anaerobic microorganisms within a biomass biodigester [2]. To date, numerous CAZyme classes and families have been discovered that target cellulose and other plant cell wall polysaccharide linkages in biofuel feedstocks [20]. Enabling technologies and software to sequence genomes/metagenomes and annotate/predict novel CAZymes have resulted in extensive literature describing new CAZymes and microorganisms for biorefinery applications.

Two areas that are pivotal for valorization of agricultural residues as viable feedstocks are: 1) to elucidate the carbohydrate composition and linkages within the plant cell wall material, and 2) to optimize enzyme, microbe, or microbial community treatments to maximize release of fermentable carbohydrates. This review will focus on recent analyses of common crop residue cell wall structures, current glycomic methods used for cell wall analysis, and in silico assessment of CAZyme function, or lack thereof, encoded within microbial communities to inform more efficient polysaccharide saccharification.

## Crop cell wall polysaccharides

**Fig. 1:**
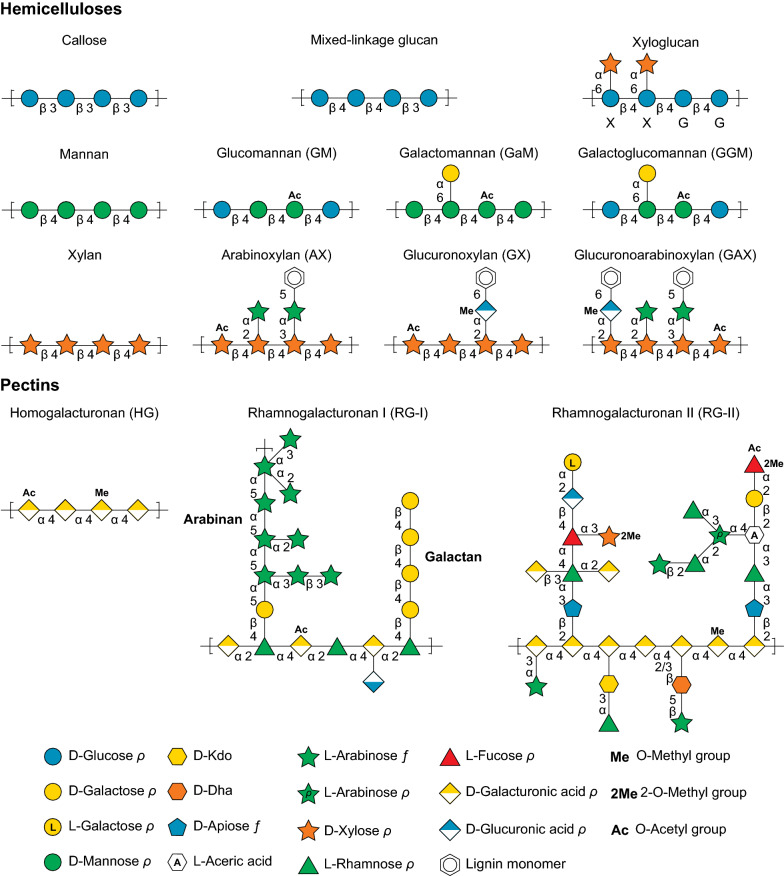
Cartoon schematic of non-cellulosic plant cell wall polysaccharides. Representative schematics chosen for xyloglucan [225], mannans and xylans [226], and pectins [24, 114]. Monosaccharide symbols follow the Symbol Nomenclature for Glycans [227]

The cell wall material of agricultural residues is comprised predominantly of cellulosic, hemicellulosic, and pectic polysaccharides, of which cellulose predominates. Cellulose is a linear chain of 4-linked *β*-D-glucopyranoses existing abundantly in the form of hydrogen-bonded, cable-like microfibrils that contain a heterogeneous mixture of crystalline and amorphous regions with a diameter ranging from 3 to 20 nm depending on cell wall type [21]. Non-cellulosic polysaccharides demonstrate great diversity in monosaccharide composition and linkage (Fig. 1). Hemicelluloses are a group of plant polysaccharides consisting mostly of 4-linked neutral sugar backbone, with or without side chains or substituent groups (*e.g.,* methyl group, acetyl group, and ferulic acids). This includes mainly xyloglucan, xylan, and heteroxylans (*e.g.,* arabinoxylan (AX), 4-*O*-methyl glucuronoxylan (GX), glucuronoarabinoxylan (GAX)), mannans, and heteromannans (*e.g.,* glucomannan (GlM), galactomannan (GaM), and galactoglucomannan (GGM)), and mixed-linkage glucans in higher plants [21, 22]. Callose is a linear 3-linked *β*-D-glucan, and although its classification of a hemicellulose is debated, it is important in higher plant cell development and responses to environmental cues [21, 23]. Pectins are a group of galacturonic acid-rich polysaccharides, including homogalacturonan (HG) and rhamnogalacturonans (RG-I and RG-II). HG has a 4-linked galacturonic acid backbone that can be 6-*O-*methyl-esterified and *O*-acetylated [21]. RG-I consists of a backbone of alternating galacturonic acids and rhamnoses and side chains of arabinan, galactan, and arabinogalactans, while RG-II is composed of a homogalacturonan backbone decorated with highly complex side chain structures built with more than 20 types of glycosidic linkages from 13 different monosaccharides [21, 24]. Aside from the wide variety of monosaccharide and linkage composition between polysaccharides, the cell wall becomes increasingly complex when considering inter- and intra-chain interactions between polysaccharides. Cellulose microfibrils commonly interact with pectin and hemicelluloses (xylans, mannans, and xyloglucan) through hydrogen bonding [25]. Pectins are also known to gel and interact with one another in the presence of calcium and boron [26]; as well, cross-linking within arabinan chains in pectins [27] and AX chains [28] by feruloyl residues has been well noted. Structural variation is complex and has been extensively studied and reviewed [21, 29]. Importantly, variations in the fine chemistry of these networks exist between plant species and at different developmental stages [30].

### Crop cell wall polysaccharide variation

Monocot (cereal crops, such as corn, wheat, and barley) and dicot plants (legumes, oilseeds, and soybeans) have similar cellulose content in primary and secondary cell walls, but differ greatly in the abundance and chemistry of hemicelluloses [31–34]. Typically, monocots contain much more heteroxylans than dicots in both the primary (20–40 vs. 5%) and secondary cell wall (40–50 vs. 20–30%) [31, 35–39]. Heteroxylans can vary greatly in their substitution patterns, effecting interactions with cellulose and lignin, and in turn, biomass recalcitrance [17, 40]. Dicots generally contain more GX, whereas monocot heteroxylans contain arabinose sidechains (AX and GAX) [18]. This difference can be observed between common agricultural crops, including canola, a dicot [41], and cereals [42]. Mixed-linkage glucans are absent in most dicots, but represent 10–30% of total cell wall content in monocots [31, 39]. This is in contrast to xyloglucan (20–35 vs. 5%) and pectins (20–25 vs. 1–5%), which are more prevalent in primary cell walls of dicots rather than monocots [31, 36, 39]. Although large differences in hemicellulose content and composition exist between monocot and dicot plants, variation can also be seen within a single group. For example, monocot heteroxylans can differ in concentration, presence of GAX or AX, and xylan substitution level or arabinose:xylose ratio [42–45]. Furthermore, variation can be seen at the species level as xyloglucan sidechains were shown to differ between canola species *B. napus* and *B. campestris* [41], between plant anatomy (*e.g.,* root vs. root hairs; sugarcane bagasse vs. straw) [46, 47], and between developmental stages in rice [48].

Plant cell wall polysaccharides are not the only structural differences observed; cross-linking between structural carbohydrates by lignin are also diverse. Lignin is a hydrophobic, polyphenolic biopolymer consisting mainly of three phenylpropanoid monomers with varying degrees of methoxylation, including p-hydroxyphenyl (H), guaiacyl (G), and syringyl (S) units [49]. Lignin increases cell wall recalcitrance by forming complex interactions with plant cell wall hemicelluloses, including heteroxylans in monocots and heteromannans in dicots [17] (Fig. 1). Lignin in monocot crops contains substantially more ferulic and ρ-coumaric acid than in dicots [31]. These components form covalent linkages with arabinose sidechains on GAX and AX; however, lignin can also be conjugated to the backbone of GGM [17, 31].

Notably, the structural diversity of plant cell wall polysaccharides and lignin polymers that exists in nature can be further augmented by common pre-treatments that cause chemical modification of cell wall polysaccharides [34]. Thus, a comprehensive understanding of plant cell wall chemistry is helpful throughout the treatment process.

## Cell wall analysis techniques

**Fig. 2:**
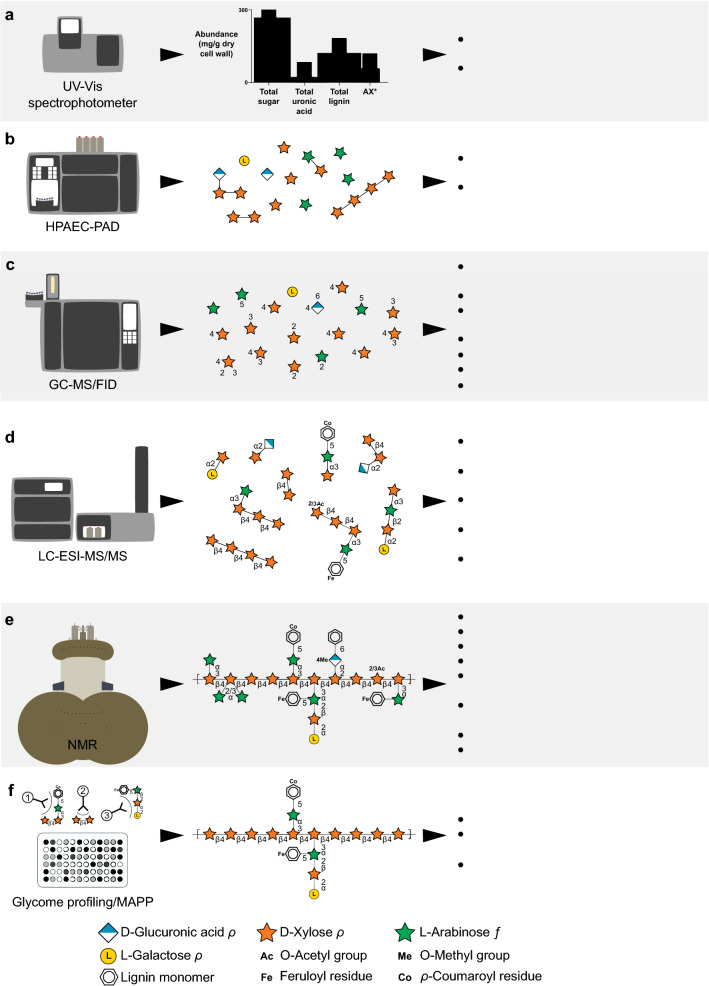
Analytical methods for total cell wall analysis. **a** UV/Vis spectrophotometer colorimetric assays. AX*: total arabinoxylan can be determined through commercially available kit; **b** HPAEC-PAD; **c** GC–MS/FID; **d** LC–ESI–MS/MS; **e** NMR; and **f** Immunological methods, such as Glycome profiling and MAPP. Corn GAX was used as a model polysaccharide to demonstrate representative structural information that could be inferred by each method [28]

Glycomic analysis of plant cell walls has seen a recent resurgence in part due to the demand for using plant biomass for biofuels [22]. These methods have improved and proven useful in elucidating the structure of native crop plant cell wall polysaccharides [50], modifications resulting from pre-treatments, and biodigester waste residues [51–53]. Glycomic analysis of lignocellulose can range from composition (*e.g.,* total sugar, total lignin, monosaccharide composition, and lignin monomer composition) to detailed structural features (*e.g.,* glycosidic linkage composition and sequences; lignin–carbohydrate interaction) with the use of advanced analytical instruments and techniques described below and summarized in Fig. 2.

### UV–Vis spectrophotometer

Colorimetric assays (Fig. 2a) can be performed using a simple UV–Vis spectrophotometer for quantification of neutral carbohydrates [54], uronic acids [55, 56], lignins [57], and substituents groups (*e.g.,* ferulate and acetate) [58–60] of whole plant cell walls prepared from agricultural residues. A broad range of enzymatic–colorimetric assay kits are commercially available (*e.g.,* Megazyme, Sigma-Aldrich) for the analysis of starch and non-starch polysaccharides, such as arabinan, AX, mixed-linkage glucan, GlM, and GaM in lignocellulosic biomass of agricultural residue.

### High-performance anion-exchange chromatography with pulsed amperometric detection (HPAEC-PAD)

HPAEC-PAD (Fig. 2b) is convenient for the identification of liberated neutral monosaccharides and uronic acids from plant residues [61]. Neutral sugars from non-cellulosic components of agricultural residue can be readily hydrolyzed by trifluoroacetic acid (TFA) into alditol acetates for analysis (*e.g.,* 2 M, 120 °C, 2 h) [22, 62]; however, sulfuric acid is normally used for the complete hydrolysis of recalcitrant crystalline cellulose in agricultural residue [22]. Methanolysis combined with TFA hydrolysis is best suited for water-soluble uronic acid-containing polysaccharides [63, 64]. Complementary to HPAEC-PAD, reverse-phase high-performance liquid chromatography coupled to ultraviolet detection (RP-HPLC–UV) with various pre- or post-column derivatization approaches (*e.g.,* 1-phenyl-3-methyl-5-pyrazolone) are available for monosaccharide analysis [65, 66]. A benefit of HPAEC-PAD is that it does not require derivatization; it is more commonly used than the RP-HPLC–UV method for monosaccharide analysis of plant residues. In addition to monosaccharide analysis, HPAEC-PAD is an important method for detecting and quantifying oligosaccharides and evaluating the purity of purified oligosaccharide samples [67, 68].

### Gas chromatography–mass spectrometry/flame ionization detection (GC–MS/FID)

GC–MS/FID (Fig. 2c) is an essential tool for the monosaccharide analysis of agriculture residues. Over the past several decades, many derivatization methods have been developed for GC–MS/FID analysis of monosaccharides [69]. Among them, the alditol acetate (AA) derivatization method is the most common [70]. Notably, a GC–MS procedure has been recently developed for comprehensive monosaccharide analysis of insoluble lignocelluloses resistant to acid hydrolysis based upon alditol acetate derivatization [71]. Glycosidic linkage analysis, normally referred to as “methylation analysis,” is a fundamental technique for structural characterization of plant cell wall polysaccharides based on GC–MS/FID analysis of the partially methylated alditol acetate (PMAA) derivatives prepared by permethylation, hydrolysis, reduction, and peracetylation of whole cell wall and fractions [22, 70, 72, 73]. Uronic acids in plant residues are converted to their corresponding 6,6-dideuterio neutral sugars before methylation analysis [74, 75]. Deuteriomethylation or ethylation is used for localizing the naturally existing *O*-methyl group during linkage analysis of cell wall polysaccharides (*e.g.,* 4-*O*-methylglucuronic acids of GX) [76–78]. The relative composition of plant polysaccharides can be estimated from the results of linkage composition by assigning glycosidic linkages to corresponding polysaccharide structures followed by summing up all the values grouped to each structure [22].

### Liquid chromatography electrospray ionization tandem mass spectrometry (LC–ESI–MS/MS)

LC–ESI–MS/MS (Fig. 2d) is most commonly used for determining the molecular mass and linkage sequence of oligosaccharides generated by partial depolymerization of cell wall polysaccharides through enzymatic and/or chemical means (*e.g.,* weak acid hydrolysis, methanolysis, acetolysis, alkaline degradation, and β-elimination) [79]. Oligosaccharides are usually purified using graphitized carbon solid-phase extraction before structural characterization by LC–ESI–MS/MS [67]. NMR and other MS techniques (*e.g.,* MALDI-tof–MS) are complimentary to LC–ESI–MS/MS for structural analysis of oligosaccharides released enzymatically or chemically from plant residues [67, 68, 80]. Recently, there has been interest in the development of LC–MS-based methods for glyosidic linkage analysis [81–85], and LC–ESI–MS/MS methods have been developed for fast monosaccharide analysis with high sensitivity [86–88]. These novel methylation-LC–MS analyses are fast and sensitive and can be used to complement current GC-based linkage analyses.

### Nuclear magnetic resonance (NMR)

Advanced structural features (*e.g*., anomeric configuration, ring forms, substituents, glyosidic linkage composition, and sequence) of polysaccharides isolated from agricultural residues can be obtained by a series of one-dimensional (1D), two-dimensional (2D) (e.g., COSY, TOCSY, HSQC, HMBC, NOESY, and ROESY), and three-dimensional (e.g., TOCSY-HSQC) solution-state NMR experiments (Fig. 2e; [89, 90]). A recently developed method involving permethylation followed by 2D ^1^H-^13^C HSQC solution-state NMR analysis can be used for polysaccharide profiling of whole cell wall [91]. A novel method for collecting 2D ^1^H-^13^C HSQC NMR spectra from non-derivatized ball-milled whole cell wall dissolved in deuterated reagents (*e.g.,* DMSO-d6/pyridine-d5) has been increasingly popular for lignocellulose characterization [49, 92–95]. Impressive progress has been made within the past decade in solid-state NMR analysis by the production of uniformly isotope-labeled plant and fungi cell wall samples by feeding ^13^CO_2_ or media containing ^13^C-glucose and ^15^N-salts, and by the introduction of ultrahigh-field (*e.g.,* 900 MHz) NMR spectrometers [40, 96, 97]. For instance, recent high-resolution multi-dimensional magic-angle spinning solid-state NMR evidence indicated that cellulose, hemicelluloses, and pectins could be associated non-covalently with the sub-nanometer scale to form an integrated network in plant primary cell walls [97]. A series of high-resolution solid-state 2D ^13^C-^13^C correlation NMR methods specifically designed for enhancing the detection of lignin aromatic signals were successfully used for the structural characterization of lignin–carbohydrate interface of plant secondary cell walls (*e.g.,* mature stems of rice, maize, and switchgrass) [98].

### Glycome profiling/microarray polymer profiling (MAPP)

Large collections (more than 200 worldwide) of cell wall glycan-directed monoclonal antibodies (mAbs) with known glycan epitope-binding specificities have allowed for the development of immunological methods for screening plant cell wall samples, termed glycome profiling (Fig. 2f; [99]). This analysis is conducted on fractionated plant cell walls using increasingly harsh chemicals, followed by an ELISA of the fractions in a 96-well plate; the results are commonly presented as a heat map [99]. Alternatively, a microarray polymer profiling (MAPP) procedure involving the integration of cell wall sequential fractionation with the generation of microarrays probed with glycan-binding mAbs or carbohydrate-binding modules (CBMs) has been developed [100]. Both immunological procedures have proved to be very useful for high-throughput screening of whole cell wall polysaccharides and their degradation products during and after bioconversion, and can be used in combination with other polysaccharide screening techniques, such as Fourier transform infrared spectroscopy-attenuated total reflectance [101–105].

## CAZymes in the production of biofuels

**Fig. 3:**
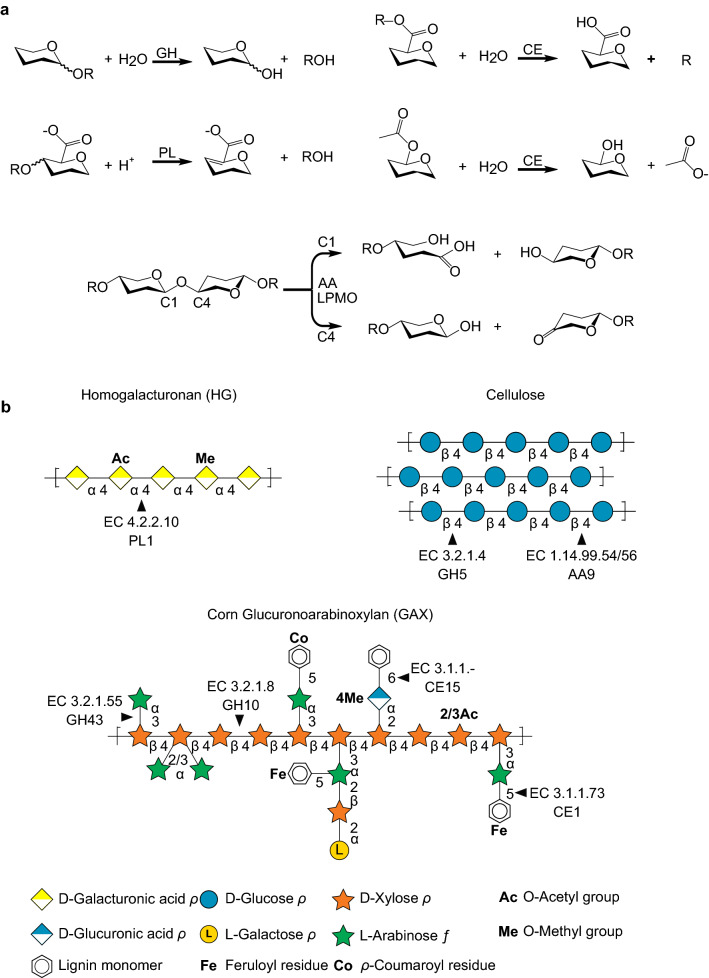
CAZyme depolymerization mechanisms and specificities. **a** Simplified reaction schematics are shown of a glycoside hydrolase (GH), polysaccharide lyase (PL), carbohydrate esterases (CEs) acetyl (top) and methyl (bottom), and the auxiliary activities (AA) of LPMOs active on C1 and C4. **b** CAZyme-targeted bonds of plant cell wall polysaccharides homogalacturonan (HG), cellulose, and corn GAX [28]) are shown, with example CAZy family and enzyme class (EC) numbers as indicated

CAZymes are classified based upon the catalytic mechanism by which they act, including glycoside hydrolases (GH) [106, 107], polysaccharide lyases (PL) [108], carbohydrate esterases (CE) [108], and auxiliary activities (AA) [109] (Fig. 3a). Each of these classes are further divided into sequence-related families.

GHs hydrolyze glycosidic bonds between carbohydrates or a carbohydrate and aglycone moiety, such as lipids or proteins [106, 107]. For most GH-mediated hydrolysis, two residues are critical for this enzymatic mechanism, a proton donor and a nucleophile/base, and results in a mechanism that either retains or inverts the anomeric configuration [106, 110]. With such diverse substrate potential existing in nature [111], it is unsurprising that GHs have been found to be active on carbohydrate polymers ranging from homopolymers, such as starch [112] and cellulose (Fig. 3b) [113], to highly branched and chemically heterogeneous substrates, such as pectins [24, 114]. At the time of publication, GHs have been classified into 168 sequence-based families in the CAZy database [115].

PLs cleave polysaccharide chains with a β-elimination reaction, resulting in a terminal hexenuronic acid [108, 110]. PLs are typically involved in the cleavage of acidic substrates, such as pectins (*e.g.,* HG; (Fig. 3B)), chondroitin, xanthan, and alginate [116]. At the time of publication, 40 different PL families have been assigned within the CAZy database [115].

CE families are currently classified into 18 different families [115]. These enzymes catalyze the de-O- or de-N-acetylation of esterified sugars through a variety of mechanisms, whereby the sugar can either act as the acid (*e.g.,* pectin methyl esters) or the alcohol (*e.g.,* acetylated xylan) (Fig. 3b; [110]). Removal of carbohydrate esters increases the access of GHs and PLs to their substrates, and therefore is an important event in the catabolism of chemically complex polysaccharides.

AAs are the most recently described CAZyme class and deploy a redox reaction to fragment structural polysaccharide and lignin substrates [109]. AAs are currently divided into 16 families, encompassing 9 families of ligninolytic enzymes, and 6 families of lytic polysaccharide monooxygenases (LPMOs), and while first [20] discovered to target chitin [117], LPMOs have demonstrated activity on common plant cell wall polysaccharides including cellulose. (Fig. 3b). Many AA enzymes are metalloenzymes, requiring copper to catalyze the digestion of lignocellulosic biomass [118, 119].

### Cellulose-active CAZymes

Cellulose is the most homogeneous and abundant source of glucose in agricultural biomass. Despite its simple *β*-1,4-linked glucose repeating structure, the crystalline higher-order structure of cellulose limits the access to cellulose-degrading CAZymes [120]. However, synergistic effects are observed when multiple enzymes are used in combination on intact cellulose, which can help overcome poor enzyme efficacy [121, 122]. Combined strategies, involving several different *exo*- and *endo*-acting GHs are used for efficient saccharification [123–126]. *Endo*-*β*-1,4-glucanases (enzyme class (EC) 3.2.1.4) cleave internal bonds within the cellulose chains and represent most enzymes used for the hydrolysis of glucosidic linkages in cellulose, while cellobiosidases (EC 3.2.1.91) processively release disaccharides from cellulose chains. Cellobiose and cellooligosaccharides released are further depolymerized by *endo*-*β*-glucosidases (EC 3.2.1.21), cellodextrinases (EC 3.2.1.74), and cellobiose phosphorylases (2.4.1.20). Cellodextrinases are preferentially active on longer substrates and hydrolyze terminal, non-reducing *β*-d-glucosyl residues from cellulose in a step-wise fashion [127].

**Fig. 4:**
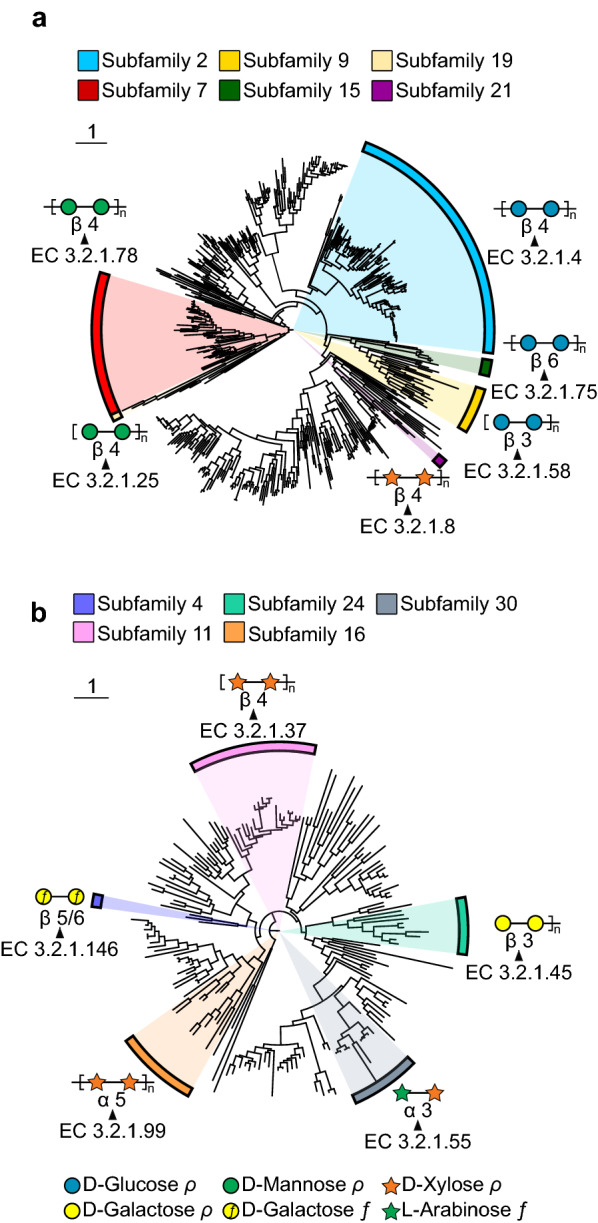
Polyspecific CAZy families GH5 and GH43. Phylogenetic trees were built using SACCHARIS [195] with characterized sequences for **a** GH5 and **b** GH43 CAZy families. Annotations were generated using ITOL [228]. Enzyme activities, for example, subfamilies, are labeled with the corresponding EC numbers, and targeted substrates are illustrated by cartoons following the Symbol Nomenclature for Glycans [227]

GH5, GH6, GH7, GH9, GH12, and GH45 CAZy families contain most cellulose-active hydrolases [115, 128, 129]. GH5 is one of the largest polyspecific GH families in the CAZy database. Once known as “cellulase family A,” it is now known to contain a variety of catalytic specificities, including *endo*-glucanase, as well as many others, including *endo*-mannosidase (EC 3.2.1.78), *endo*-xylanase (EC 3.2.1.8), and *endo*-*β*-1,6-glucosidase (EC 3.2.1.75). As such, the GH5 family has been further subdivided into sequence-related subfamilies to better classify conserved specificities [130] (Fig. 4a). The GH6 family consists solely of *endo*-glucanases and cellobiohydrolases, which also compose most of the GH7 family. GH9 is the second largest family of cellulase enzymes, comprised primarily of *endo*-glucanases. *Endo*-glucanases are found in the GH12 family, among xyloglucan *endo*-transglycosidase and xyloglucan *endo*-hydrolase activities. Finally, GH45 family members function as *endo*-glucanases; however, some are specific to xyloglucan.

Weak *endo*-glucanase activity was seen in the GH61 and CBM33 family. However, both these families are now understood to be LPMOs, which target cellulose through oxidative cleavage. GH61 has been reclassified as AA9 [131], while CBM33 has been reclassified as AA10 and is known to possess enzymes active on cellulose or chitin [117].

### Hemicellulose- and pectin-active CAZymes

Due to the abundance of xylan in plant cell walls, there has been a concerted effort to understand xylan and heteroxylan digestion by *endo*-*β*-1,4-xylanases (EC 3.2.1.8), *β*-1,4-xylosidases (EC 3.2.1.37), arabinan *endo*-*α*-1,5-l-arabinanases (EC 3.2.1.99), and non-reducing end *α*-l-arabinofuranosidases (EC 3.2.1.55).

GH10 and GH11 predominantly contain *endo*-*β*-1,4-xylanases, and enzymes from these families work synergistically to break down xylan and heteroxylan. GH11s are active on xylans at least seven sugars in length, while GH10s are better suited to the hydrolysis of xylosyl linkages close to arabinosyl-substitutions [132]. As well, in highly substituted wheat bran AX, GH10 xylanases are able to accommodate arabinose decorated xylose residues, whereas GH11 xylanases do not [133].

AX is a large component of monocot hemicellulosic polysaccharides and thus a common substrate for arabinofuranosidases and arabinanases. GH43 is a polyspecific family divided into many subfamilies [134] (Fig. 4b) and contains many *α*-l-arabinofuranosidases and *α*-l-arabinanases active on AX. Arabinofuranosidases have been classified based on substrate determinants [132]:: (1) type A, active on pNP-*α*-l-arabinofuranosides and short arabinooligosaccharides; (2) type B, active on short oligosaccharides and longer polysaccharides, such as arabinan and AX; and (3) AX arabinofuranohydrolases. Recent studies have shown that rumen fungi are adept at producing GH43 enzymes for the breakdown of complex hemicelluloses, and these enzymes may represent the most abundant fungal glycoside hydrolases for these reactions [135].

CE enzymes (*e.g.,* acetyl xylan esterase EC 3.1.1.72, feruloyl esterase EC 3.1.1.73, CE families 1 through 7) can facilitate accessibility of hydrolytic enzymes to their substrates, as large modifications, substitutions, and cross-linking of carbohydrate residues impede enzymatic catalysis. For example, corn bran is highly recalcitrant to enzymatic digestion [136, 137], likely due to ferulate cross-links within AX [138], but the inclusion of acetyl xylan esterases (CE1) and feruloyl esterases (CE1), alongside xylanases (GH10), xylosidases (GH3), and arabinofuranosidases (GH43, GH51) significantly increased the release of total monomeric xylose [28]. The cooperation between the different enzyme activities of CEs and GHs may be necessary for the complete hydrolysis of heavily modified hemicellulosic and pectic polysaccharides. Interestingly, there is some recent evidence to suggest that LPMOs are also active on xylans and xyloglucans and contribute to the large array of catalytic strategies evolved to dismantle these complex substrates [139].

### Modifying plant genetics to reduce recalcitrant residues

Glycosyltransferases (GTs) are responsible for the synthesis of structural polysaccharides, storage polysaccharides, and other complex glycans [140]. The formation of glycosidic bonds involves the transfer of a carbohydrate moiety from sugar donors to acceptor molecules [110], and cascading glycosylation by downstream GTs results in increasingly complex carbohydrates. For example, biosynthesis of plant pectic polysaccharides requires hundreds of glycosyltransferases to produce the extensive variety of glycosidic linkages and adducts [141]. Genetic manipulation of these biological processes can reduce the number of recalcitrant residues in the plant cell wall [17], namely cellulosic [142] and hemicellulosic [18] biomass. Initial attempts have been made as an alternative to enzymatic treatment, such as the downregulation of GT8 family pectin biosynthetic genes in switchgrass which leads to decreased lignocellulose and pectin cross-linking, thereby reducing the recalcitrance of biomass [143, 144].

### Strategies for CAZyme-catalyzed digestion of lignocellulosic biomass

Interactions between cellulose, hemicellulose, pectin, and lignin leads to a complex network that is highly recalcitrant to enzymatic deconstruction. Studies have begun to look at the hydrolysis of these interactions by enzymes, such as AA family LPMOs [131]. Additionally, AA lignin-modifying enzyme families may have a role; laccases, manganese peroxidases, and lignin peroxidases all potentially contribute to the modification of cross-links and subsequent delignification, exposing the underlying polysaccharides for further modification by GH and CE enzymes [145]. Along with feruloyl esterase, CE15 glucuronyl esterases also contribute to the disassembly of lignin–carbohydrate complexes via the cleavage of ester bonds between alcohol and 4-*O*-methyl-glucuronoyl moieties of lignin and xylan, respectively (Fig. 3b) [146]. Degradation of lignocellulosic biomass has improved using cellulolytic enzyme cocktails [147] and combining lignin-active enzymes with polysaccharide-specific enzymes may be the best strategy for the optimal digestion of complex lignocellulose [148]. Tailoring the mixture to the agricultural residues of interest, and the specific polysaccharides and glycosidic linkages, may be optimal for converting these biological residues into valuable products.

Lignocellulose deconstruction in bioethanol production employs extensive heat treatments to expose biomass for efficient enzymatic attack, often at temperatures above 55 °C [127]. Thus, enzymes are often sourced from thermophilic microbes as they are the most likely to retain properties beneficial for bioprocessing. For example, a GH5 endo-glucanase from *Talaromyces emersonii* was found to have optimal activity at pH 4.8 and 80 °C, but retains activity for 15 min at temperatures up to 100 °C [149]. Furthermore, non-enzymatic processes that decrease the crystallinity of cellulose typically involve low pH, organic solvents, chemical and oxidative reagents, and detergents [127]. Some enzymes, such as two thermostable cellulases of *Melanocarpus albomyces*, are more active on crystalline cellulose than amorphous cellulose [150]. These conditions and enzymatic properties need to be taken into consideration when selecting enzymes for the treatment of biomass residues.

## -Omic and bioinformatic approaches to elucidate CAZyme function

**Fig. 5:**
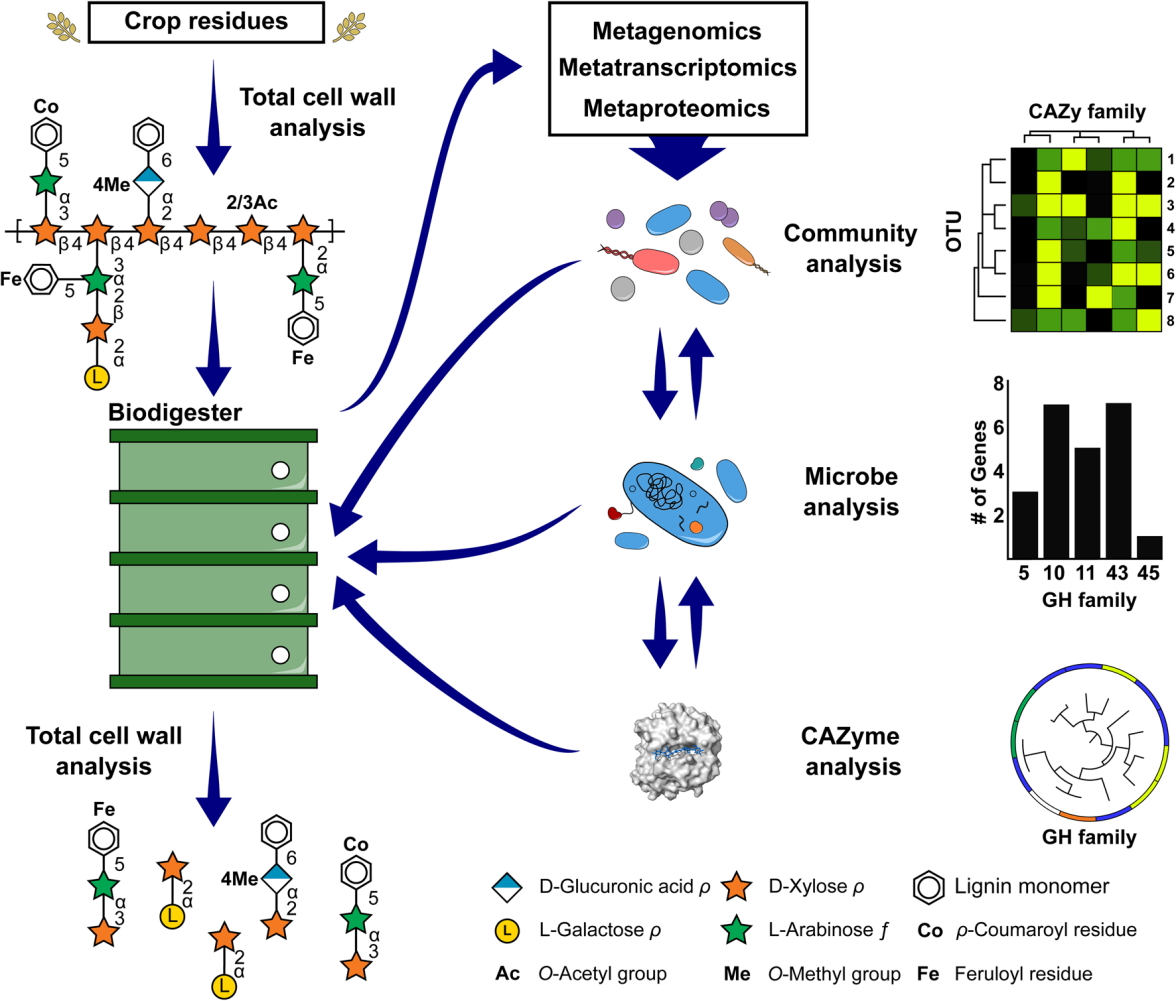
Combinatorial assessment of cell wall structure and investigation of microbial CAZyme function. The integration of analytical methods can be implemented to provide a comprehensive experimental workflow to improve bioconversion of agriculture residues. Crop residues can be studied prior to or after processing using total cell wall analysis. Information on the structure of waste residues can be compared to starting material to determine recalcitrant structures that are limiting the efficiency of bioconversion. The microbial ecosystem of biodigesters can be studied using -omics techniques, such as metagenomics, metatranscriptomics, and metaproteomics, to define the structure and function at the community, microbe, and CAZyme levels. Information gathered using these techniques can inform optimized conditions or identify lacking catalytic functions in the reaction cascade. Microbial communities, microorganisms, and CAZymes can be deployed back into production processes to augment inefficent or absent catalytic reactions and improve biofuel production. Surface representation of enzyme structure (white) was generated using PyMOL [229] (PDB ID: 2CKR), with cellotetrose ligand illustrated in sticks (blue)

Extensive research has been invested toward identifying CAZymes, microorganisms, and microbial communities that are capable of saccharifying lignocellulose to reduce the cost and increase the yield of biofuel production. Commonly, organisms selected for fermentation (*e.g., Saccharomyces cerevisiae*) lack the ability to metabolize lignocellulose [151]. Fungi and bacteria, including the well-studied *T. reesei* and *Clostridium* spp., are used to produce lignocellulosic CAZymes [152, 153], as they can secrete large quantities of endogenous cellulolytic CAZymes (*i.e., endo*-glucanases, *exo*-glucanases, glucosidases [152], and LPMOs [154]). These CAZymes have greatly increased the efficiency of ethanol production, but the cost of producing and purifying enzymes can make the process economically untenable [19]. To provide affordable solutions for optimized lignocellulose degradation, it is common to bioprospect microbial ecosystems of biodigester systems involved in plant biomass saccharification to identify lignocellulose-degrading microorganisms and their endogenous CAZymes. Promising microbes and/or CAZyme targets have been discovered in crop soil [155], compost [156], wastewater sludge [157], and herbivorous animal microbiomes [158, 159]. Significantly, the anaerobic environment of the ruminant digestive tract and the termite hindgut has led to the discovery of novel species and microorganisms, including the obligate anaerobic fungi phylum *Neocallimastigomycota* in cattle rumen [160] and lignocellulosic microorganisms found in and cultivated by termites [159, 161]. Microbial analysis of anaerobic environments is of particular interest to the bioethanol and biogas industries due to the parallels that exist between these environments. Moreover, biogas biodigesters are enriched with lignocellulose-degrading organisms as they are optimized for biomass metabolism. Microorganisms and/or CAZymes identified within biodigesters can be used as supplements to further increase the valorization of biodigester feedstocks (Fig. 5). Crop residues, including corn stover [162], barley straw [163], rice straw [164], and wheat straw [165], are commonly used as biodigester feed stocks. However, microbial community composition can vary greatly between systems depending on pH, temperature, and feed substrates [2, 166].

Lignocellulose-metabolizing microorganisms can exhibit varied growth conditions depending on their taxonomy and the environment they were isolated from [167], making the cultivation of organisms and discovery of novel CAZymes encoded within their genomes difficult. However, with the recent advances in -omics technologies and decreases in associated costs, the study of complex communities has become more accessible. Metagenomics [168–170], metatranscriptomics [163, 166, 171], and metaproteomics [162, 172, 173] have demonstrated the utility of -omics technologies for the discovery of lignocellulose microorganisms and CAZymes. When combined with reference genomes or metagenomes, metatranscriptomics and metaproteomics allow for accurate functional assignment of genes and proteins, respectively [174]. Recent advances in metagenomic sequencing and contig binning have ushered in a new era of metagenomic-assembled genomes, allowing for increased understanding of microbial function within and between microbial ecosystems [175, 176]. For example, a large-scale metagenomic study demonstrated the diversity of species between anaerobic digesters and the importance of generating metagenomic assembled genomes to study and standardize a core and accessory digester microbiome, allowing for efficient optimization of biogas production [177]. Metagenomics and associated software for annotation and functional prediction have also aided in the assembly of eukaryotic genomes in complex environments, which overcomes the historical challenge of sequencing eukaryotic genomes [178]. Genomic and metagenomic databases have rapidly expanded and will continue to do so as the affordability and accessibility of second- and third-generation sequencing technologies increase. Unfortunately, subsequent biochemical characterization of annotated genes has been unable to keep pace with sequencing data. Therefore, accurate and automated annotation of these sequences has become a priority for streamlining CAZyme discovery.

### CAZyme annotation and curation

Wide-ranging guidelines have been proposed for unifying how metagenomic studies are performed, covering aspects from sample collection and metagenomic binning [179, 180] to standards for metagenomically generated genomes [175]. Additionally, there are renowned software pipelines for the prediction and annotation of prokaryotic and eukaryotic genes, including PROKKA [181], RAST [182], MAKER2 [183], AUGUSTUS [184], and the NCBI online annotation platforms [185]. Annotation platforms, such as COG [186], SEED [187], Pfam [188], and KEGG [189], have also been instrumental for predicting gene function. However, these platforms are not specialized for CAZyme annotation, nor are they designed to differentiate between the rapidly expanding lists of CAZyme families.

The CAZy database was launched in 1999, and is the single source for CAZyme curation [20]. In addition, it provides links to relevant publications and other online resources, such as CAZypedia [190] and the polysaccharide utilization loci (PUL) database PULDB [191]. These resources have enabled other external platforms to assist with CAZyme discovery and characterization. For example, the CAZyme annotation tool dbCAN [192] provides hidden Markov models (HMMs) generated from the CAZy database to facilitate user sequence annotation. dbCAN identifies sequence boundaries to improve prediction accuracy, creating profile HMMs based on homologous sequence alignments. Alternatively, the CAZyme analysis toolkit [193], currently unmaintained, implements Pfam-defined profile HMMs which were recently shown to identify > 98% of GHs in the CAZy database [194]. These profile HMMs provide valuable protein domain prediction, especially helpful in determining boundaries in multi-modular CAZymes and/or attached CBM modules [195], and are currently used by an expanding list of pipelines and software tools [195–197]. However, it should be noted that due to differing thresholds between profile HMMs, there may be discrepancies between Pfam and dbCAN annotations when compared to those of CAZy [20].

The addition of subfamily designations to large, polyspecific families in the CAZy database and the subsequent profile HMMs generated by dbCAN have greatly improved functional prediction of novel sequences for CAZy families GH5 [130], GH13 [198], GH16 [199], GH30 [200], and GH43 [134]. However, there are still inherent limitations with family- and subfamily-based classifications. While members with CAZy families possess the same fold and catalytic mechanisms, assignment of a sequence to a CAZy family is not necessarily definitive of enzyme specificity. Functional differences between members of the same subfamily and polyspecific families without subfamily delineations convolute prediction of CAZyme activity. As well, sequence-based CAZyme prediction is hampered by the low abundance of characterized sequences in the database and variability in substrate libraries used to biochemically characterized enzymes. In this regard, a standardized approach using similar substrates and kinetic parameters to report rate would be beneficial. Fortunately, there is a growing list of novel software packages designed to aid in the annotation (PULpy [201], DRAM [202], and dbCAN-PUL [203]), curation (dbCAN-PUL [203]) and high-resolution phylogeny (SACCHARIS [195], CUPP [196]) of uncharacterized CAZymes.

Both PULpy and DRAM software packages use profile HMMs sourced from both dbCAN and Pfam to identify CAZymes. PULpy focuses heavily on identifying polysaccharide utilization loci (PULs) within metagenomes, demonstrated in ruminants [169], and DRAM extrapolates CAZyme annotation to predict carbohydrate utilization of identified taxonomic units. Recently, dbCAN-PUL was developed for the curation of PULs by substrate, taxonomy, and characterization method. The repository can also be downloaded and used as a database to BLASTX against novel CAZymes. Alternatively, SACCHARIS is a pipeline that streamlines identification and phylogenetic analysis of CAZyme sequences. Sequences collected from the CAZy database, as well as user input sequences, are trimmed to the predicted catalytic domain using dbCAN, aligned [204], and a best-fit Newick tree is generated [205–207] (Fig. 4). SACCHARIS is a real-time software which enables the functional prediction of CAZymes based upon tree topologies generated using the current state of knowledge [80, 208, 209]. The Conserved Unique Peptide Patterns (CUPP) downloadable software uses peptide pattern recognition to find conserved peptide motifs within CAZyme families to develop strict CUPP groups or subfamilies, and a recent web server allows for annotation of user sequences [210]. CUPP has been used to elucidate sequence function in pectin and alginate lyase families [211, 212], as well as using fungal CAZyme secretomes to predict fungal phylogenies [213]. Together with -omics-based technologies, CAZyme prediction tools will aid in the interpretation of sequence datasets at the microbe, community, and gene level. Ultimately, this interpretation is necessary to inform CAZyme discovery and characterization, which can be used to improve biofuel production (Fig. 5).

## Glycomic and multi-omic integration

Methods to resolve the fine chemistry of biofuel feedstocks and to optimize the valorization of feedstocks through discovery of microorganisms and CAZymes have led to significant advances in biofuel production. Combining these approaches will help unlock further solutions for optimizing the synthesis and saccharification of recalcitrant biomass. Comparative genomics of plant cell wall biosynthetic loci is a complementary approach to glycomics to help illuminate the structural diversity of cell walls that exists between species [30]. Plants employ a wide variety of CAZymes to synthesize, remodel, and saccharify plant cell walls during growth and development [214, 215], and -omics can be used to identify functional orthology between cell wall biosynthetic genes [216]. A multi-tiered approach that includes plant cell wall profiling and CAZyme gene mining has been proposed to better understand cell wall variability between plant species [215]. Recently, CAZyme phylogeny and characterization have been supplemented with analytical methods to investigate acetyl xylan synthesis [217], and variable expression of xylan synthesis glycotransferases between species [218]. This combinatory approach of glycomics and -omics will prove to be crucial in the generation of “designer” biofuels [18].

Additionally, the combination of glycomics and multi-omics provides direct and indirect insights into plant cell wall structure and saccharification of recalcitrant biomass. The use of glycomics in conjugation with -omics has been used to determine the activity and saccharification products of CAZymes in a variety of fields (e.g., human health [219], soil health/carbon sinks [220], novel enzyme discovery [221], and recalcitrant biomass saccharification [222]). However, this strategy is challenged by the complexityof host dynamic microbial ecosystems, CAZymes, and complex carbohydrate structures. Although many researchers have expanded their focus to study CAZymes from anaerobic digesters, leading to an expansion of -omic datasets [157, 177], and likewise, perform glycomic research on biomass saccharification in anaerobic digesters or animal digestive organs [51, 52], there are few studies which combine these tools to fully understand the complexity of anaerobic digesters. Using metatranscriptomics, researchers determined CAZyme expression profiles in *Aspergillus niger* grown on wheat straw with different pre-treatment methods [223]. The pre-treated wheat straw and resulting growth cultures were analyzed using HPAEC-PAD to determine which CAZymes induced the differential expression patterns between pre-treatment methods. Furthermore, the combination of MAPP, linkage analysis, and metagenomics has recently been used to determine the CAZymes responsible for the digestion of non-soluble polysaccharides in chickens—an approach highly portable to anaerobic digesters [224]. As the field of biofuels progresses, a multi-disciplinary approach will be needed to fine-tune and standardize methods to optimize production, as diversity in microorganisms in combination with feedstocks and feedstock pre-treatments can drastically alter saccharification and fermentation efficiencies.

## Conclusion

Improving biofuel production from crop residues is a promising avenue for increasing the value of agricultural waste streams. Although there has been substantial progress made toward understanding the cell wall structure of crop residues, structural variation that exists between plant species and tissues, and chemical modifications resulting from pre-treatments impacts their efficient use in biofuel production. State-of-the-art glycomic methods can be used to provide a high-resolution picture of plant cell wall structure in crop residues, and previous studies have emphasized the importance of using this structural knowledge to detect inefficiencies in biomass fermentation [52, 53] (Fig. 5). Intensified research of crop residue cell wall structure and composition will be informative for designing tailored approaches for individual plant sources. As well, with the advancement of -omics technologies, availability of sequence datasets, and bioinformatic tools developed to interpret metadata, it has become more feasible to discover and deploy novel CAZymes biocatalysts, saccharolytic microbial species, and microbial communities tuned for specific crop residues (Fig. 5). Together, elucidation of biomass cell wall structure and innovations in CAZyme technologies will help streamline future efforts to improve the efficiency of biofuel production, helping unlock the energy potential of agricultural crop waste streams and next-generation biofuel feedstocks.

## Data Availability

Data sharing is not applicable to this article as no datasets were generated or analyzed during the current study.

## Abbreviations

CAZyme: Carbohydrate-active enzyme
PM: Particulate matter
Kdo: Ketodeoxyoctonic acid
Dha: 3-Deoxy-lyxo-heptulosaric acid
AX: Arabinoxylan
GX: Glucuronoxylan
GlM: Glucomannan
GaM: Galactomannan
GGM: Galactoglucomannan
HG: Homogalacturonan
RG-I: Rhamnogalacturonan I
RG-II: Rhamnogalacturonan II
GAX: Glucuronoarabinoxylan
HPAEC-PAD: High-performance anion-exchange chromatography with pulsed amperometric detection
TFA: Trifluoroacetic acid
RP-HPLC–UV: Reverse-phase high-performance liquid chromatography coupled to ultraviolet detection
GC–MS/FID: Gas chromatography–mass spectrometry/flame ionization detection
PMAA: Partially methylated alditol acetates
LC–ESI–MS/MS: Liquid chromatography electrospray ionization tandem mass spectrometry
NMR: Nuclear magnetic resonance
MAPP: Microarray polymer profiling
mAb: Monoclonal antibodies
CBM: Carbohydrate-binding module
GH: Glycoside hydrolase
PL: Polysaccharide lyase
CE: Carbohydrate esterase
AA: Auxiliary activities
EC: Enzyme class
LPMO: Lytic polysaccharide monooxygenase
GT: Glycosyltransferase
PUL: Polysaccharide Utilization Loci
HMM: Hidden Markov model
SACCHARIS: Sequence Analysis and Clustering of CarboHydrate Active enzymes for Rapid Informed prediction of Specificity
CUPP: The Conserved Unique Peptide Pattern
