# Correction to: Combined whole cell wall analysis and streamlined in silico carbohydrate‑active enzyme discovery to improve biocatalytic conversion of agricultural crop residues

**DOI:** 10.1186/s13068-021-01888-z

**Published:** 2021-02-08

**Authors:** Jeffrey P. Tingley, Kristin E. Low, Xiaohui Xing, D. Wade Abbott

**Affiliations:** 1 Lethbridge Research and Development Centre, Agriculture and Agri-Food Canada, 5403-1st Avenue South, Lethbridge, AB T1J 4B1 Canada; 2 Department of Biochemistry, University of Lethbridge, Lethbridge, AB T1K 6T5 Canada

## Correction to: Biotechnol Biofuels (2021) 14:16 10.1186/s13068-020-01869-8

Following publication of the original article [1], the authors noticed an error in the figures. It was noticed that due to typesetter error and file conversion, incorrect versions of the figures were published. The corrected Figs. 1, 2, 3, 4, 5 are given below.

**Fig. 1:**
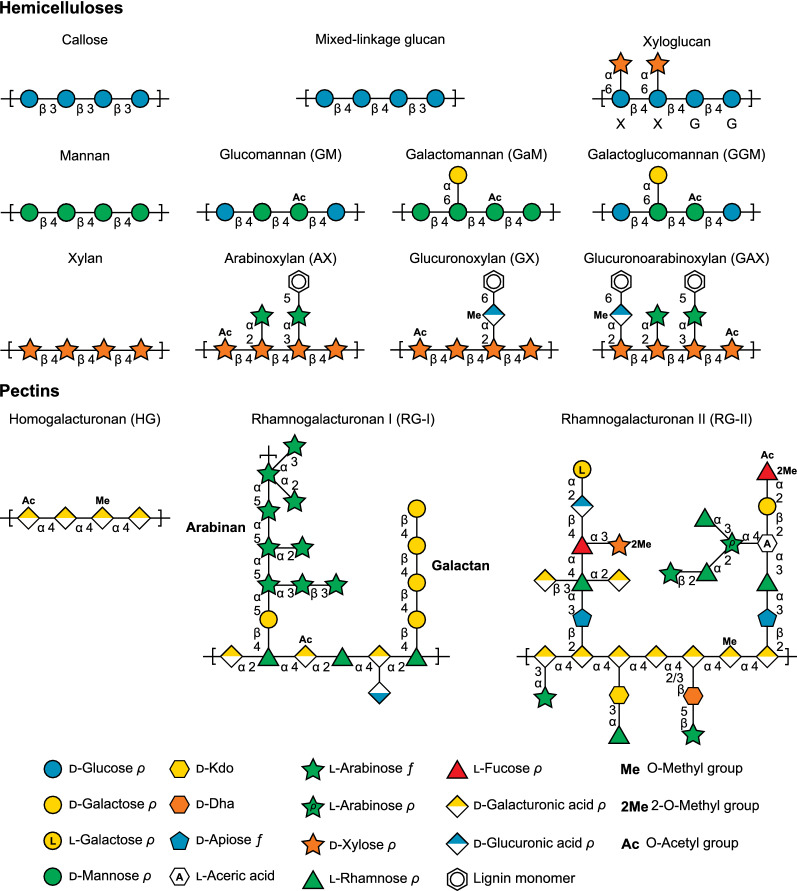
Cartoon schematic of non-cellulosic plant cell wall polysaccharides. Representative schematics chosen for xyloglucan [225], mannans and xylans [226], and pectins [24, 114]. Monosaccharide symbols follow the Symbol Nomenclature for Glycans [227]

**Fig. 2:**
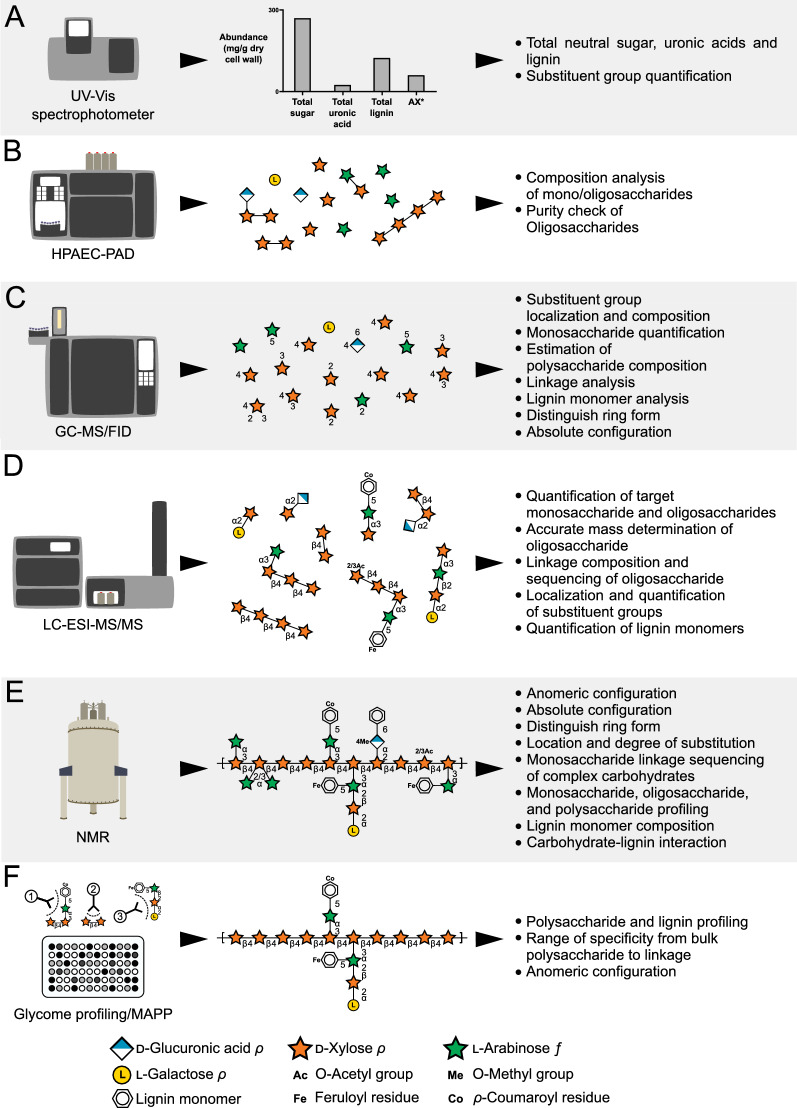
Analytical methods for total cell wall analysis. a UV/Vis spectrophotometer colorimetric assays. AX*: total arabinoxylan can be determined through commercially available kit; b HPAEC-PAD; c GC–MS/FID; d LC–ESI–MS/MS; e NMR; and f Immunological methods, such as Glycome profiling and MAPP. Corn GAX was used as a model polysaccharide to demonstrate representative structural information that could be inferred by each method [28]

**Fig. 3:**
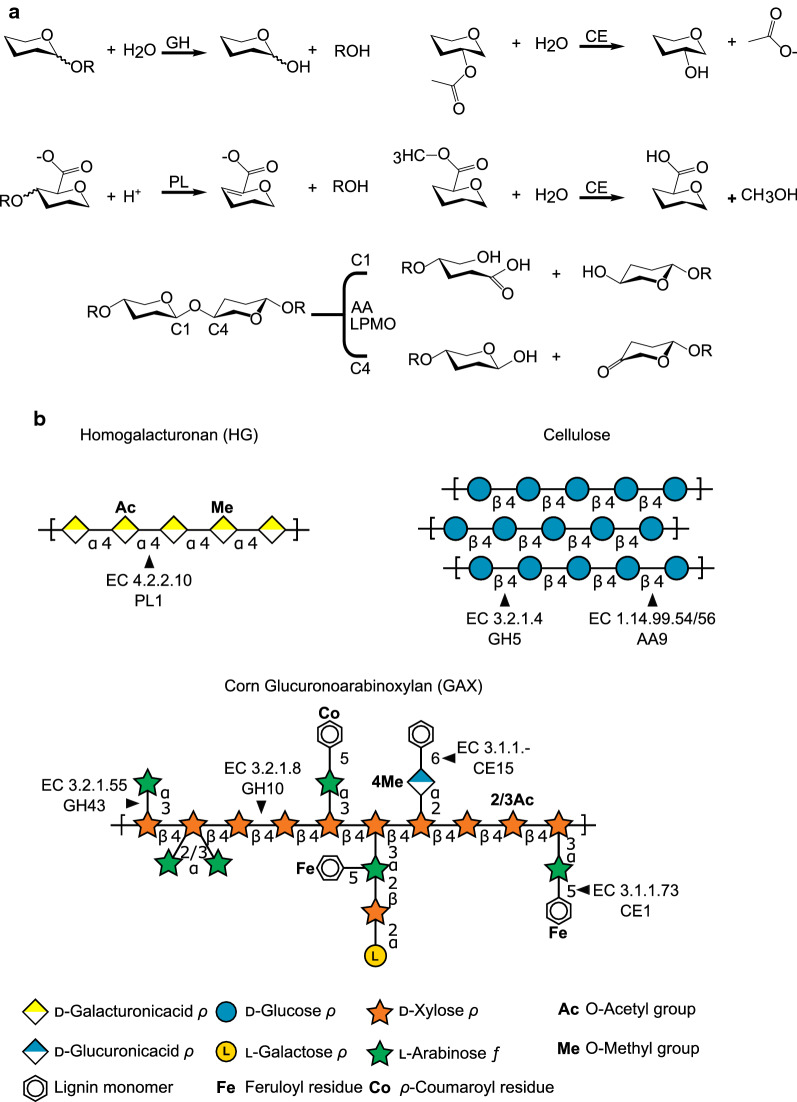
CAZyme depolymerization mechanisms and specificities. **a** Simplified reaction schematics are shown of a glycoside hydrolase (GH), polysaccharide lyase (PL), carbohydrate esterases (CEs) acetyl (top) and methyl (bottom), and the auxiliary activities (AA) of LPMOs active on C1 and C4. **b** CAZyme-targeted bonds of plant cell wall polysaccharides homogalacturonan (HG), cellulose, and corn GAX [28]) are shown, with example CAZy family and enzyme class (EC) numbers as indicated

**Fig. 4:**
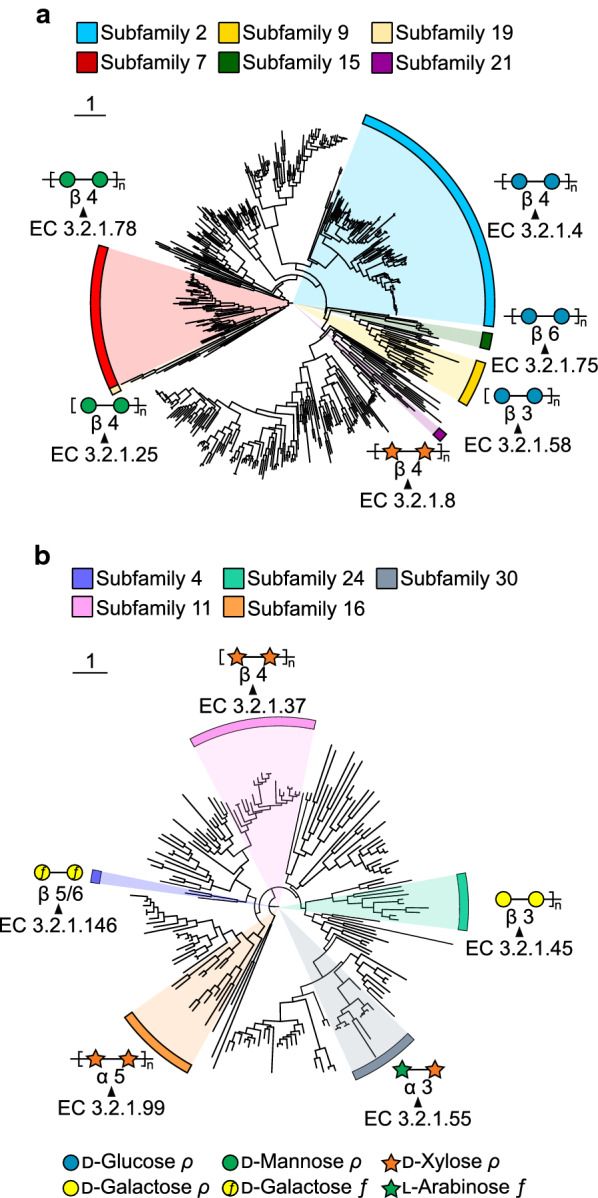
Polyspecific CAZy families GH5 and GH43. Phylogenetic trees were built using SACCHARIS [195] with characterized sequences for a GH5 and b GH43 CAZy families. Annotations were generated using ITOL [228]. Enzyme activities, for example, subfamilies, are labeled with the corresponding EC numbers, and targeted substrates are illustrated by cartoons following the Symbol Nomenclature for Glycans [227]

**Fig. 5:**
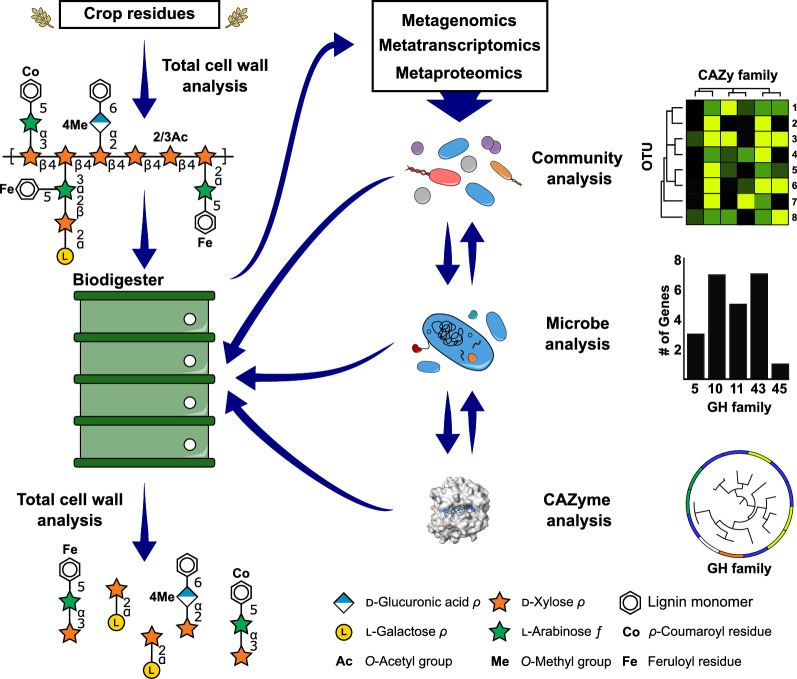
Combinatorial assessment of cell wall structure and investigation of microbial CAZyme function. The integration of analytical methods can be implemented to provide a comprehensive experimental workflow to improve bioconversion of agriculture residues. Crop residues can be studied prior to or after processing using total cell wall analysis. Information on the structure of waste residues can be compared to starting material to determine recalcitrant structures that are limiting the efficiency of bioconversion. The microbial ecosystem of biodigesters can be studied using -omics techniques, such as metagenomics, metatranscriptomics, and metaproteomics, to define the structure and function at the community, microbe, and CAZyme levels. Information gathered using these techniques can inform optimized conditions or identify lacking catalytic functions in the reaction cascade. Microbial communities, microorganisms, and CAZymes can be deployed back into production processes to augment inefficent or absent catalytic reactions and improve biofuel production. Surface representation of enzyme structure (white) was generated using PyMOL [229] (PDB ID: 2CKR), with cellotetrose ligand illustrated in sticks (blue)

The original article [1] has been updated.
